# Synthesis of a tetrazine–quaterthiophene copolymer and its optical, structural and photovoltaic properties

**DOI:** 10.1007/s10853-019-03551-3

**Published:** 2019-04-03

**Authors:** Astrid-Caroline Knall, Sebastian Franz Hoefler, Manuel Hollauf, Ferula Thaler, Sven Noesberger, Ilie Hanzu, Heike Ehmann, Mathias Hobisch, Stefan Spirk, Shuguang Wen, Renqiang Yang, Thomas Rath, Gregor Trimmel

**Affiliations:** 1 Institute for Chemistry and Technology of Materials (ICTM), NAWI Graz, Graz University of Technology, Stremayrgasse 9, 8010 Graz, Austria; 2 Anton Paar GmbH, Anton Paar Straße 20, 8054 Graz, Austria; 3 Institute of Paper, Pulp and Fibre Technology, Graz University of Technology, Inffeldgasse 23, 8010 Graz, Austria; 4 CAS Key Laboratory of Bio-based Materials, Qingdao Institute of Bioenergy and Bioprocess Technology, Chinese Academy of Sciences, Qingdao, 266101 China

## Abstract

Herein, we report the synthesis of a novel, tetrazine-based conjugated polymer. Tetrazines have the benefit of being strong electron acceptors, while little steric hindrance is imposed on the flanking thiophene rings. Conversion of a suitably substituted nitrile precursor led to 3,6-bis(5-bromo-4-(2-octyldodecyl)thiophen-2-yl)-1,2,4,5-tetrazine (2OD-TTz). Palladium-catalyzed copolymerization of 2OD-TTz with a bithiophene monomer yielded an alternating tetrazine–quaterthiophene copolymer (PTz4T-2OD). The polymer PTz4T-2OD showed an optical band gap of 1.8 eV, a deep HOMO energy level of − 5.58 eV and good solubility. In combination with the non-fullerene acceptor ITIC-F, solar cells with power conversion efficiencies of up to 2.6% were obtained.

**Electronic supplementary material:**

The online version of this article (10.1007/s10853-019-03551-3) contains supplementary material, which is available to authorized users.

## Introduction

Organic photovoltaics are gathering a tremendous amount of attention in the scientific community. In particular, fullerene-free organic solar cells have sparked interest amongst researchers since novel donor and acceptor structures have extended the range of possible combinations to tune the device performance to an optimum. By combining the right conjugated polymer donor and non-fullerene acceptor (NFA) partners, power conversion efficiencies (PCEs) have reached values of up to 15.6% in single-junction solar cells [1–3] and 17% in tandem devices [4], while the quest for suitable materials on both sides is still ongoing. Thus, NFA-based solar cells have already outperformed PCBM ([6, 6]-phenyl-C_71_-butyric acid methyl ester)-based devices. It is not only possible to prepare NFAs in large quantities and good purity, but even more importantly, they can be tailored more easily than fullerenes to match the energy levels with the donor in order to improve the device performance [5–11]. Large conjugated frameworks are a structural feature of the most potent NFAs, one example being 3,9‐bis(2‐methylene‐(3‐(1,1‐dicyanomethylene)‐indanone))‐5,5,11,11‐tetrakis(4‐hexylphenyl)‐dithieno[2,3‐*d*:2′,3′‐*d*′]‐*s*‐indaceno[1,2‐*b*:5,6‐*b*′]dithiophene (ITIC, [12]). ITIC-based systems, in particular those with fluorinated ITIC derivatives, have been successfully used to obtain devices with PCEs of up to 14% [1, 13, 14].

However, also on the polymer side, research is focusing on novel conjugated donor–acceptor (D–A) polymers with alternative donor and acceptor building blocks aiming at polymer structures with improved optoelectronic properties. Ideally, these building blocks are straightforward in terms of synthetic accessibility and can be easily modified to tailor the polymer properties. One example for such acceptor building blocks are tetrazines which can be prepared in a conceptually simple synthesis from the corresponding nitriles, meaning that side chains and substituents can be introduced in a straightforward manner. Due to their strong electron-accepting character, tetrazines have been frequently applied in Diels–Alder reactions with inverse electron demand (iEDDA) [15]. Because of the lack of hydrogen atoms on the tetrazine ring, steric hindrance is reduced allowing the flanking thiophene rings to arrange in a coplanar fashion.

Liu et al. [16] combined a series of different acceptor units with a quaterthiophene donor moiety. The observed high PCEs (around 10%) and fill factor (FF) values (above 75%), in particular in thicker films, which are beneficial for potential industrial-scale production, were achieved by controlling the polymer–fullerene morphology. The films exhibited small polymer domains with high crystallinity and purity, which is beneficial for high charge carrier mobilities. This was achieved by attaching (2-octyl-)dodecyl side chains in the 4-position on the flanking thiophene rings with respect to the acceptor unit, while bithiophene was used as donor. In addition to fullerene-based devices, NFA-based solar cells with promising PCE values of up to 9% have been reported using PffBT4T‐2OD (poly[(5,6‐difluoro‐2,1,3‐benzothiadiazol‐4,7‐diyl)‐*alt*‐(3,3′″‐di(2‐octyldodecyl)‐2,2′;5′,2″;5″,2′″‐quaterthiophen‐5,5′′′‐diyl)]) as polymeric donor in the absorber layer [17, 18].

Also for bis(thienyl)tetrazine-based polymers, placing the alkyl chains in 4-position instead of the 3-position on the flanking thiophenes leads to increased coplanarity due to less steric hindrance [19, 20]. In combination with a cyclopenta[2,1-*b*:3,4-*b*′]dithiophene (CPDT) monomer, this type of tetrazine-based monomer led to the so far highest observed PCE of around 5.5% using PCBM as acceptor [21, 22], while 5.1% were reached for benzo[1,2-*b*:4,5-*b*’]dithiophene (BDT)-type tetrazine copolymers [23, 24]. Alternative polymer structures led to less efficient solar cells; copolymers with dithieno[3,2-*b*:2′,3′-*d*]silole (DTS) reached PCE values of up to 4.2% [25], whereas the best-performing fluorene [20] and carbazole [20] copolymers resulted in devices with efficiencies of up to 0.8% and around 2%, respectively.

Based on these results, a tetrazine with two flanking thiophene units, substituted with 2-octyldodecyl side chains in the 4-position, was identified as a promising structure to be copolymerized with bithiophene, leading to the alternating tetrazine–quaterthiophene copolymer poly[(1,2,4,5-tetrazin-3,6-diyl)-alt-(3,3′′′-di(2-octyldodecyl)-2,2′;5′,2′′;5′′,2′′′-quaterthiophen-5,5′′′-diyl)] (PTz4T-2OD). The objective of this study is the synthesis and characterization of PTz4T-2OD as well as the investigation of its photovoltaic potential in polymer–NFA solar cells. ITIC-F (3,9-bis(2-methylene-(3-(1,1-dicyanomethylene)-6-fluoro-indanone))-5,5,11,11-tetrakis(4-hexylphenyl)-dithieno[2,3-*d*:2′,3′-*d*′]-*s*-indaceno[1,2-*b*:5,6-*b*′]dithiophene [26]) was selected as NFA since its energy levels and optical properties match well with those of PTz4T-2OD.

## Materials and methods

All reagents and solvents were purchased from commercial sources (Sigma-Aldrich or TCI) with reagent-grade quality and were used as received. All solvents were dried using a column-based solvent purification system except CH_2_Cl_2_, which was dried by distillation over CaH_2_ as drying agent. ITIC-F was synthesized according to Refs. [14, 26].

### Monomer synthesis

The tetrazine monomer 2OD-TTz (3,6-bis(5-bromo-4-(2-octyldodecyl)thiophen-2-yl)-1,2,4,5-tetrazine) was synthesized in analogy to previously published protocols [27–29]. Details for the synthetic procedures of 2OD-TTz can be found in the supporting information.

### Polymer synthesis

For the copolymerization, 2OD-TTz (100.16 mg, 0.1037 mmol, 1 eq), 5,5′-bis(trimethylstannyl)-2,2′-bithiophene (51.05 mg, 0.1038 mmol, 1 eq), Pd_2_(dba)_3_ (2.28 mg, 2.49 µmol, 0.024 eq) and P(o-tol)_3_ (3.15 mg, 1.04 µmol, 0.1 eq) were placed in a 10-mL glass tube and dissolved in chlorobenzene (4 mL). The mixture was degassed with nitrogen for 30 min. Afterwards, the tube was sealed and placed into a conventionally heated synthesis reactor (Monowave 50 from Anton Paar GmbH, Graz, Austria) and subjected to the following temperature program: ramp to 180 °C (for 10 min), 180 °C (30 min holding time). The dark blue mixture was added dropwise to cold methanol to precipitate a blue polymer. The crude product was purified by Soxhlet extractions in acetone, cyclohexane and chloroform. The majority of the product was dissolved in the chloroform fraction. This solution was concentrated to a volume of about 5 mL and precipitated into cold methanol to obtain the purified polymer. Yield: 82.2 mg, blue-violet powder. ^1^H-NMR (*δ*, 20 °C, CDCl_3_, 500 MHz): 8.06 (br, 2H), 7.23 (br, 4H), 2.82 (br, 4H), 2.11 (br, 2H), 1.93–0.55 (m, 76H), ^13^C-NMR (*δ*, 20 °C, CDCl_3_, 125 MHz): 160.90, 138.66, 138.48, 134.64, 134.23, 127.98, 124.44, 124.11, 38.80, 37.16, 34.27, 33.52, 33.38, 32.09, 31.59, 30.07, 29.71, 29.39, 26.57, 22.82, 22.72, 14.14, FT-IR (cm^−1^): 3062, 2956, 2924, 2854, 1548, 1504, 1455, 1349, 1067.

### Characterization techniques

Nuclear magnetic resonance (NMR) spectroscopy was performed on Bruker Avance 300 MHz and Varian Inova 500 MHz spectrometers. Deuterated solvents were obtained from Cambridge Isotope Laboratories Inc. Spectra were referenced against the residual proton signals of the solvent according to the literature [30]. Peak shapes are specified as follows: s (singlet), bs (broad singlet), d (doublet), dd (doublet of doublets), t (triplet), q (quadruplet) and m (multiplet). FT-IR spectroscopy measurements were acquired on a Bruker Alpha FT-IR spectrometer in transmission using undoped Si-wafers as substrates or in ATR-mode using an ALPHA Platinum ATR single reflection diamond ATR module. Silica gel 60 F254 and aluminium oxide 60 F254 (both from Merck) on aluminium sheets were used for thin-layer chromatography. Visualization was done under UV light or by dipping into an aqueous solution of KMnO_4_ (0.1 wt%). MALDI-TOF mass spectrometry was performed on a Micromass TofSpec 2E time-of-flight mass spectrometer. The instrument was equipped with a nitrogen laser (*λ* = 337 nm, operated at a frequency of 5 Hz) and a time lag focusing unit. Ions were generated just above the threshold laser power. Positive ion spectra were recorded in reflection mode with an accelerating voltage of 20 kV. The spectra were externally calibrated with a polyethylene glycol standard. Analysis of data was done with MassLynx-Software V3.5 (Micromass/Waters, Manchester, UK). High-temperature gel permeation chromatography (GPC) measurements were performed on an Agilent Technologies PL-GPC220 instrument with 1,2,4-trichlorobenzene as eluent with a PLgel MIXED-B LS 300 × 7.5 mm column and a refractive index detector (1.00 mL min^−1^, 150 °C, 200 µL injection volume). Thermogravimetric analysis measurements were performed on a Netzsch STA 449 C thermogravimetric analyser using aluminium oxide crucibles in the temperature range between 20 and 550 °C with helium as purge gas (flow rate: 50 mL min^−1^) and a heating rate of 10 K min^−1^. Absorption spectra of the polymer thin films were recorded on a Shimadzu UV-1800 UV–Vis spectrophotometer in the range of 300–1000 nm. Absorption coefficients were determined from thin films deposited by spin coating from chlorobenzene solutions. Cyclic voltammetry measurements were carried out in acetonitrile using a three-electrode set-up consisting of a platinum (Pt) mesh (counter electrode), an Ag/Ag^+^ reference electrode [31] and an indium tin oxide (ITO)-coated glass substrate (15 × 15 mm, 15 Ω/sq, Kintec) coated with a thin film of PTz4T-2OD as working electrode. The reference electrode was calibrated against a ferrocene–ferrocenium solution (Fc/Fc^+^) in deoxygenated and anhydrous acetonitrile using tetrabutylammonium hexafluorophosphate (Bu_4_NPF_6_, 0.1 M) as supporting electrolyte and a scan rate of 50 mV s^−1^. The ionization potential (IP) and the electron affinity (EA) were calculated from the onset of the oxidation and the reduction potential (*E*_ox_, *E*_red_) of the polymer considering the energy level of Fc/Fc^+^ to be − 4.8 eV below the vacuum level via

1 $$ E_{\text{IP}} = - \left[ {\left( {E_{\text{ox}} - E_{{{\text{ox}}\left( {\text{Fc}} \right)}} + 4.8} \right)} \right]\; {\text{eV}} $$

2 $$ E_{\text{EA}} = - \left[ {\left( {E_{\text{red}} - E_{{{\text{ox}}\left( {\text{Fc}} \right)}} + 4.8} \right)} \right] \;{\text{eV}} $$

where *E*_ox(Fc)_ is the oxidation potential of ferrocene [32]. 2D-GIWAXS measurements of polymer thin films spin-coated on silicon substrates were performed on an Anton Paar SAXSpoint 2.0 system equipped with a Dectris 2D EIGER R 1 M hybrid photon counting detector with 75 µm^2^ pixel size and using Cu K_α_ radiation at 50 kV and 1 mA, which was point-collimated using automated scatterless slits. The incidence angle was set to 0.12°, and the exposure time was 10 × 120 s. A spin-coated silver behenate film was used for the angular calibration. Atomic force microscopy (AFM) measurements were performed on an Anton Paar Tosca™ 400 atomic force microscope in tapping mode using Al-coated cantilevers (ARROW-NCR, NanoWorld AG) with a resonance frequency of 285 kHz and a force constant of 42 N m^−1^. All measurements were acquired at room temperature under ambient conditions. All calculations and image processing were done with Tosca™ analysis software (V7.4.8341, Anton Paar). Surface profilometry measurements were performed on a Bruker DektakXT stylus surface profiling system equipped with a 12.5-µm-radius stylus tip in order to determine the layer thickness of the thin-film samples. Line scans were recorded over a length of 1000 µm, with a stylus force of 3 mg, and a resolution of 0.33 µm pt^−1^. Layer thickness values were derived from two-dimensional surface profiles using Vision 64 software (Bruker).

### Solar cell fabrication

Pre-patterned ITO-coated glass substrates were cleaned by sonication in 2-propanol (40–50 °C, 60 min) and oxygen plasma treatment (FEMTO, Diener Electronic, 3 min). For inverted bulk-heterojunction solar cells, ZnO thin films were derived from a sol–gel reaction of a zinc oxide precursor solution consisting of zinc acetate dihydrate (0.5 g, 2.3 mmol) in 2-methoxyethanol (5 mL) using ethanolamine (150 mL, 2.5 mmol) as the stabilizer [33]. The zinc oxide precursor solution was vigorously stirred overnight under ambient conditions for the hydrolysis reaction, followed by filtration through a 0.45-μm polytetrafluoroethylene (PTFE) syringe filter before spin coating (4000 rpm, 30 s). The ZnO films were annealed under ambient conditions (150 °C, 15 min) to achieve layer thicknesses in the range of 30–40 nm. PTz4T-2OD was dissolved in chlorobenzene at 90 °C, blended with ITIC-F in a donor–acceptor ratio of 1:1 by weight (16 mg mL^−1^ total concentration), and spin-coated onto preheated substrates to obtain a layer thicknesses of about 150 nm. A molybdenum(VI) oxide anode interfacial layer (10 nm, deposition rate: ca. 0.1–0.4 Å s^−1^) and a silver anode (100 nm, 0.2–1.0 Å s^−1^) were deposited by thermal evaporation under reduced pressure (ca. 10^−5^ mbar) through a shadow mask, defining the active area (9 mm^2^). For bulk-heterojunction solar cells in normal device configuration, poly(3,4-ethylenedioxythiophene)–poly(styrenesulfonate) (PEDOT–PSS, Heraeus Clevios P VP AI 4083) was filtrated through a 0.45-µm polyvinylidene difluoride (PVDF) syringe filter and spin-coated onto the pre-cleaned ITO substrates under ambient conditions, followed by annealing (120 °C, 20 min) to obtain layer thicknesses of ca. 40 nm. The absorber layer was processed using the same procedure as described above. An aluminium cathode (100 nm, 0.3–8.0 Å s^−1^) was deposited by thermal evaporation under reduced pressure (ca. 10^−5^ mbar) through a shadow mask, defining the active area (9 mm^2^).

### Solar cell characterization

Current density–voltage (*J*–*V*) curves were recorded under illuminated and dark conditions in an inert atmosphere using a Keithley 2400 source meter and a Dedolight DLH400D metal halide lamp (1000 W m^−2^), which was calibrated with a standard reference silicon solar cell (Fraunhofer ISE). Photovoltaic characteristic parameters were determined from the *J*–*V* curves under illumination and averaged over five devices, unless otherwise stated. Series (*R*_S_) and shunt (*R*_SH_) resistance values were extracted from the *J*–*V* curves under illumination. External quantum efficiency (EQE) measurements were performed under ambient conditions using an Amko MuLTImode 4-AT monochromator equipped with a 75-W xenon lamp (LPS 210-U, Amko), a lock-in amplifier (Stanford Research Systems, Model SR830) and a Keithley 2400 source meter. The monochromatic light was chopped at a frequency of 30 Hz. The EQE spectra were measured in the wavelength range of 350–1000 nm (increment: 10 nm). The measurement set-up was spectrally calibrated with a reference photodiode (Newport Corporation, 818-UV/DB).

## Results and discussion

### Monomer synthesis

**Scheme 1:**
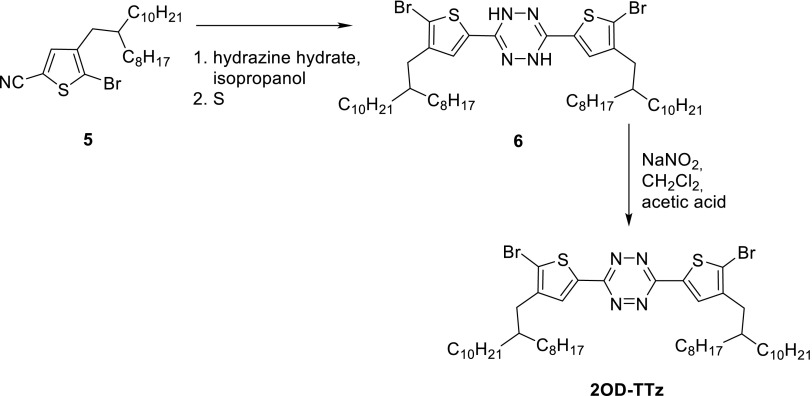
Synthesis of 2OD-TTz (see supporting information for detailed synthetic procedures)

3,6-Bis(5-bromo-4-(2-octyldodecyl)thiophen-2-yl)-1,2,4,5-tetrazine (**2OD-TTz**) was synthesized from 5-bromo-4-(2-octyldodecyl)thiophene-2-carbonitrile **5**. In order to avoid any traces of monosubstituted tetrazine monomer causing the formation of shorter polymer chains, the tetrazine formation step was performed last in the synthetic sequence. In order to obtain **5**, 3-bromothiophene was subjected to a Kumada coupling reaction with 2-octyldodecyl magnesium bromide leading to 3-(2-octyldodecyl)thiophene [27], followed by bromination with *N*-bromosuccinimide (NBS) resulting in 2-bromo-3-(2-octyldodecyl)thiophene. Subsequently, this compound was deprotonated followed by quenching with DMF to introduce an aldehyde functionality which was subsequently converted to the corresponding nitrile **5** [28, 29]. The synthetic procedures for **5** including the intermediates **1–4** are described in detail in the supporting information. From **5**, the monomer was prepared using Pinner conditions with sulphur as catalyst. Due to the large hydrophobic 2-octyldodecyl moiety, the standard solvent (ethanol) had to be replaced with isopropanol. The intermediately formed dihydrotetrazine was isolated as a crude product and directly converted to the corresponding tetrazine using sodium nitrite and acetic acid, as shown in Scheme 1.

### Polymer synthesis and characterization

**Scheme 2:**
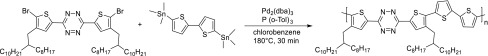
Synthesis of PTz4T-2OD

PTz4T-2OD (poly[(1,2,4,5-tetrazin-3,6-diyl)-alt-(3,3′′′-di(2-octyldodecyl)-2,2′;5′,2′′;5′′,2′′′-quaterthiophen-5,5′′′-diyl)]) was synthesized via a Stille cross-coupling polymerization of **2OD-TTz** and 5,5′-bis(trimethylstannyl)-2,2′-bithiophene using Pd_2_(dba)_3_ as catalyst, as shown in Scheme 2 [34].

One drawback of microwave-assisted polymerization is the risk of local overheating (“hot spots”) which can be observed in monomode microwave reactors (due to a higher local field density) and, in particular, at later stages of the polymerization procedure where higher viscosity of the reaction solution can potentially cause the magnetic stirring to fail. Since tetrazines are sensitive towards thermal decomposition, a conventionally heated synthesis reactor, which allows monitoring of the internal reaction temperature and permits reaction pressures of up to 20 bar, was employed.

**Figure 1:**
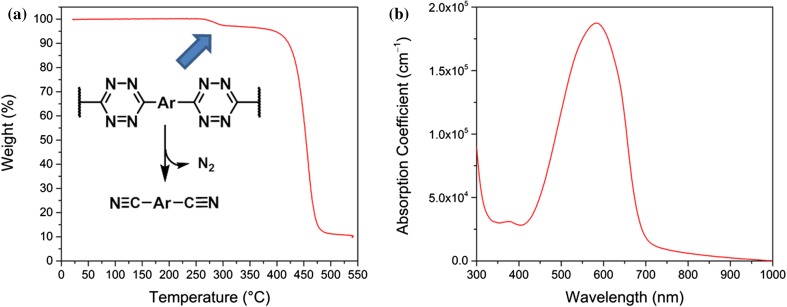
**a** Thermogravimetric analysis curve of PTz4T-2OD—the blue arrow marks the decomposition step of the tetrazine ring—and **b** absorption spectrum of a PTz4T-2OD thin film

The resulting conjugated D–A polymer **PTz4T-2OD** consists of a 1,2,4,5-tetrazine acceptor moiety and four thiophene units partially substituted with branched aliphatic side chains as donor building block. The conjugated polymer exhibits good solubility in common organic solvents such as chloroform, chlorobenzene and *ortho*-dichlorobenzene. In the FT-IR spectrum of the polymer, characteristic bands for both the tetrazine (1504 cm^−1^) and the quaterthiophene (1067 cm^−1^) units are observed (Figure S5) [35]. High-temperature gel permeation chromatography (GPC) measurements gave a number-average molecular weight (*M*_n_) of 24.1 kDa, a weight-average molecular weight of 59.3 kDa (*M*_w_) and a dispersity *Đ*_M_ of 2.46 (Table 1, Figure S6). Thermogravimetric analysis (TGA) under helium atmosphere demonstrates good thermal stability of PTz4T-2OD up to 288 °C. At this temperature, the tetrazine rings in the backbone are known to decompose to the corresponding quaterthiophene dinitriles [19, 22] and nitrogen is released which accounts for the observed mass loss of around 3% (considering a molar weight of 1000 g mol^−1^ for the repeating unit and 28 g mol^−1^ for N_2_). Previously, a yield of more than 90% has been reported for this reaction [19]. The remaining organic moieties further decompose at a decomposition temperature (*T*_d_) of approx. 460 °C (Fig. 1a).

**Table 1:** Molecular weight and thermal properties of PTz4T-2OD

*M*_n_ (kDa)	*M*_w_ (kDa)	Dispersity *Đ*_M_	*T*_d_ (°C)
24.1	59.3	2.46	288 (first step), 460 (second step)

Figure 1b and Table 2 show the absorption spectrum of a PTz4T-2OD thin film and the corresponding optical properties, respectively. PTz4T-2OD exhibits a broad absorption peak with a maximum at 583 nm together with a small peak at ca. 377 nm. The optical absorption coefficient *α* at the absorption maximum is approx. 1.9 × 10^5^ cm^−1^. The optical band gap (*E*_g_^opt^) was determined from the onset of the thin-film absorption spectrum to be 1.80 eV.

**Table 2:** Optoelectronic properties of PTz4T-2OD

*λ*_max,film_ (nm)	*α* at *λ*_max_ (10^5^ cm^−1^)	*λ*_onset,film_ (nm)	*E*_g_^opt^ (eV)	E_IP_, HOMO^a^/LUMO^b^ (eV)	E_EA_^a^ (eV)
583	1.87	688	1.80	− 5.58/− 3.78	− 3.10

^a^Determined via cyclic voltammetry

^b^Calculated via HOMO + E_g_^opt^

Cyclic voltammetry was performed to determine the highest occupied molecular orbital (HOMO) energy level of PTz4T-2OD using ferrocene–ferrocenium (Fc/Fc^+^) as external standard. From the cyclic voltammetry data (Figure S7), an ionization potential of − 5.58 eV and an electron affinity of – 3.10 eV were determined, giving an electrochemical energy gap of 2.48 eV. The optical band gap (1.80 eV) is significantly lower. Setting the value of the ionization potential equal to the HOMO energy level and using the optical band gap, a LUMO energy value of − 3.78 eV was calculated. The corresponding data are summarized in Table 2. The difference between the optical and electrochemical band gap most likely resides in the very different conditions in which these two determinations were carried out. First, it is known that the solvents, in which chemical species are dissolved, may influence strongly the position of the HOMO and LUMO levels. Although the PTz4T-2OD was prepared as a film on ITO, it is very likely that some swelling of the polymer in acetonitrile occurs. For optical band gap determination there are no solvents in which the polymer film is immersed. Second, as this polymer is a donor it is expected to undergo a much faster oxidation reaction than a reduction reaction. This is indeed clear from the cyclic voltammogram in Fig. S7; the kinetics of reduction are probably much slower than the kinetics of oxidation. This will introduce an overpotential, and thus the reduction potential used to estimate the electron affinity will be shifted to lower values. Third, although we use a supporting electrolyte we cannot be sure that all ohmic drops are negligible. While they will certainly not be very high due to the reasonable conductivity of the ITO and the thin polymer film used, they might add up with the other two effects and contribute to the increased value of the electrochemically determined energy gap.

### Molecular packing and film morphology

**Figure 2:**
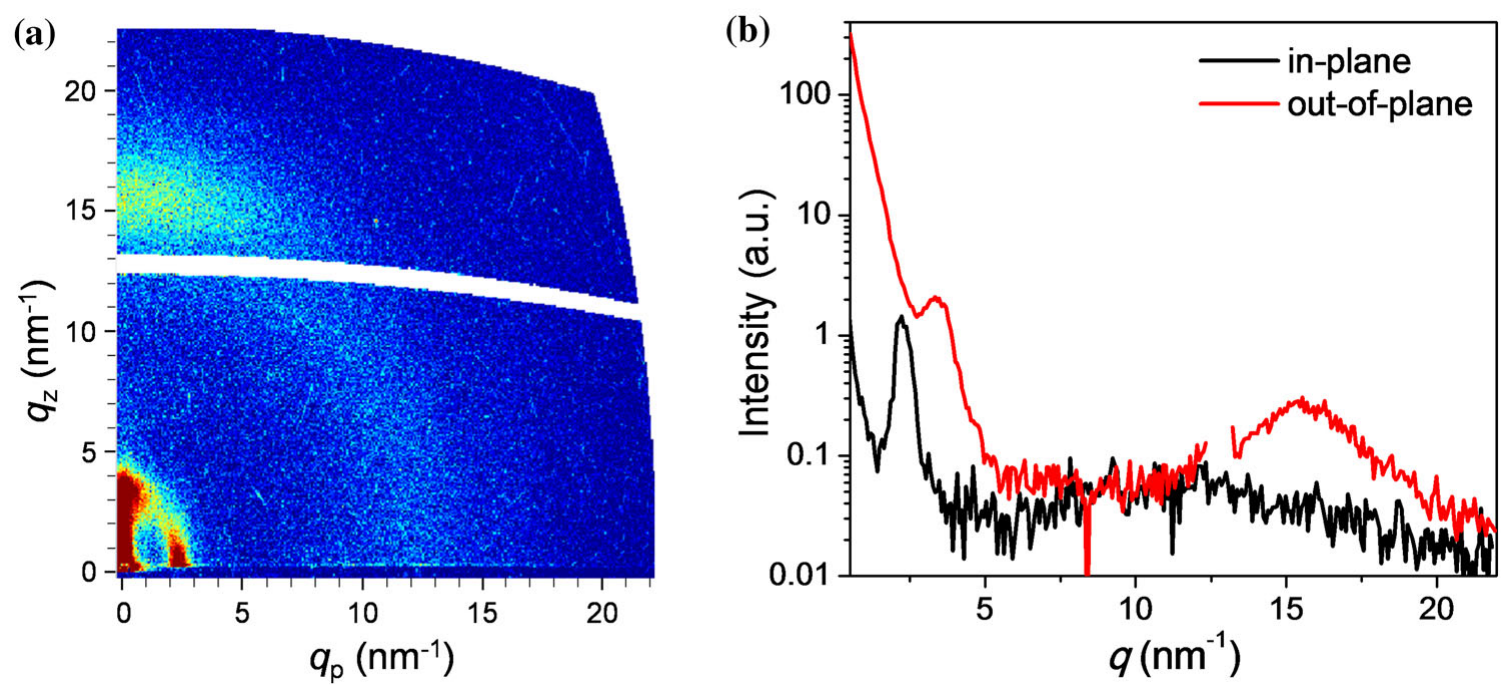
**a** 2D-GIWAXS patterns and **b** scattering profiles for the in-plane and out-of-plane directions

Moreover, two-dimensional grazing incidence wide-angle X-ray scattering (2D-GIWAXS) measurements of thin films of PTz4T-2OD were performed to gain information about the crystallization behaviour and molecular packing (Fig. 2). The GIWAXS image reveals a weak ring-like feature at *q* = 15.5 nm^−1^, which is more pronounced in the out-of-plane direction. This suggests a preferred face-on packing orientation of the polymer chains in the film, and the *d*-spacing of approx. 0.40 nm is indicative of *π*–*π* stacking. In addition to the lamellar diffraction peak at *q* ~ 2.3 nm^−1^ (interlamellar distance of approx. 2.7 nm) in the in-plane direction, which is expected for a preferential face-on orientation with respect to the substrate, a further intense area is found in the out-of-plane direction, indicating a certain isotropy in the molecular packing. The crystalline coherence length (CCL), determined from the lamellar diffraction peak in the in-plane direction, is 12 nm.

**Figure 3:**
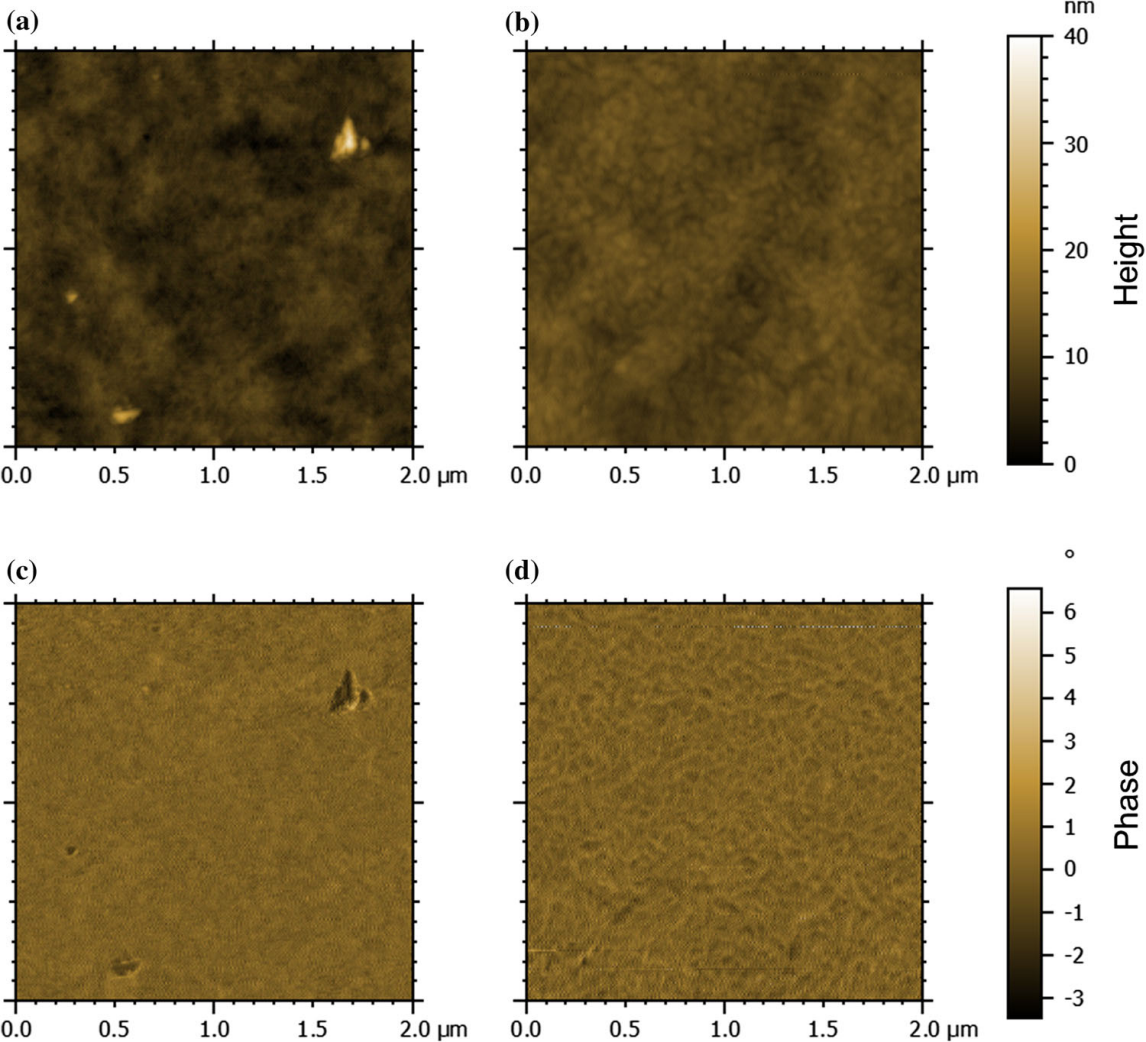
AFM topography and phase images of a pristine PTz4T-2OD film (**a**, **c**) and a PTz4T-2OD–ITIC-F blend (**b**, **d**) on a glass/ITO/ZnO substrate. The image sizes are 2 µm × 2 µm

The AFM topography image (Fig. 3a) of the polymer film spin-coated from a chlorobenzene solution reveals a very smooth surface morphology, and a very low surface roughness with an *S*_q_ value of 1.5 nm is observed. For the application as absorber layer in organic solar cells, the polymer is blended with ITIC-F. The morphology of the blend film appears similar to the pristine polymer film, and the surface roughness is essentially the same (*S*_q_ = 1.5 nm). Furthermore, the phase contrast in the blend film is very low due to the rather similar chemical composition of PTz4T-2OD and ITIC-F in the blend (Fig. 3c, d), which makes it difficult to draw any distinct conclusions about phase separation based on the AFM phase images.

### Photovoltaic performance

PTz4T-2OD was investigated in bulk-heterojunction non-fullerene organic solar cells in inverted (indium tin oxide (ITO, ca. 140 nm)/ZnO (ca. 30–40 nm)/PTz4T-2OD–ITIC-F (ca. 150 nm)/MoO_3_ (10 nm)/Ag (100 nm)) and normal device configurations (ITO (ca. 140 nm)/PEDOT–PSS (ca. 40 nm)/PTz4T-2OD–ITIC-F (ca. 120 nm)/Al (100 nm)). A schematic representation of the device architectures and the corresponding energy level diagrams are given in Figure S8A and B. In addition to the well matching energy levels of PTz4T-2OD and ITIC-F, these compounds have complementary absorption properties, as can be seen in Figure S9. For the preparation of the solar cells, a PTz4T-2OD–ITIC-F weight ratio of 1:1 was used, as this ratio gave the best results in preliminary experiments.

**Figure 4:**
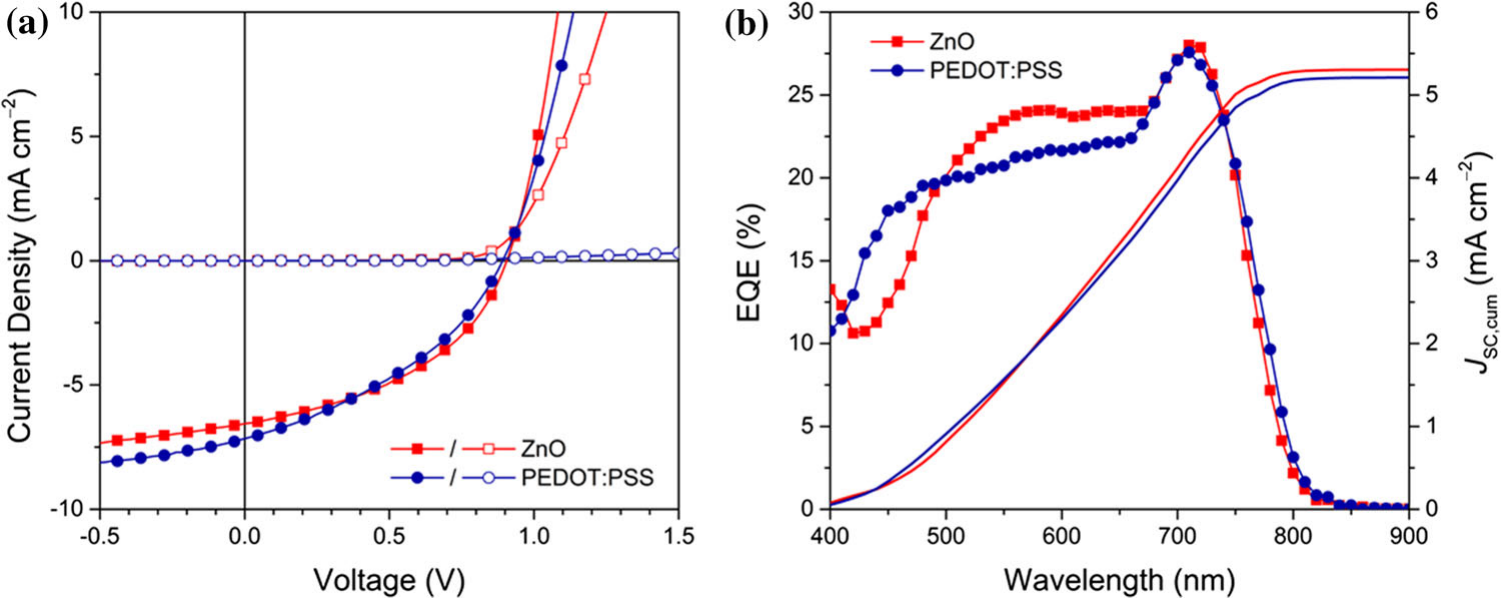
**a***J*–*V* curves of PTz4T-2OD–ITIC-F non-fullerene organic solar cells in inverted (ZnO, red) and normal (PEDOT–PSS, blue) device configurations; **b** EQE spectra of PTz4T-2OD–ITIC-F solar cells in inverted and normal architectures plotted together with the cumulated *J*_SC_

Table 3 and Fig. 4a show the photovoltaic performance parameters and the *J*–*V* curves measured under illuminated and dark conditions. In both device architectures, comparably high open-circuit voltages (V_OC_s) of around 0.9 V and short-circuit current density (*J*_SC_) values between 6 and 7 mA cm^−2^ were obtained. In particular, the FF values remained low. For example, FFs of approx. 43% were obtained in the inverted device architecture and in the normal configuration only FFs of 37% could be realized. The slightly higher *R*_S_ and the significantly lower *R*_SH_ in the normal device architecture might originate from the absence of a hole-blocking layer between the absorber layer and the Al-electrode. Overall, solar cells with PCEs of up to 2.6% could be realized in the inverted architecture and maximum PCEs of 2.35% were reached with solar cells prepared in normal architecture.

**Table 3:** Photovoltaic performance parameters of PTz4T-2OD–ITIC-F solar cells

Device architecture	*V*_OC_ (V)	*J*_SC_ (mA cm^−2^)	FF (%)	PCE (%)	*R*_S_ (Ω cm^2^)	*R*_SH_ (kΩ cm^2^)
Inverted, ZnO^a^	0.89 ± 0.02	6.31 ± 0.40	42.6 ± 2.6	2.41 ± 0.31	34.4 ± 5.0	0.57 ± 0.15
Best cell	0.91	6.55	43.7	2.59	31.9	0.43
Normal, PEDOT–PSS	0.87 ± 0.01	7.05 ± 0.11	37.5 ± 0.4	2.25 ± 0.08	43.4 ± 1.3	0.33 ± 0.06
Best cell	0.89	7.16	37.7	2.35	43.7	0.32

^a^Averaged over four devices

As already mentioned above, the best solar cell performance was obtained with an absorber layer thickness of about 150 nm. By using thinner absorber layers, the FF could be slightly improved, however, only at the expense of a reduced *J*_SC_ (Table S1). Additional annealing of the absorber layers did not lead to improved solar cell characteristics. The corresponding results are shown in Figure S10. Moreover, initial shelf life tests of the solar cells revealed promising results (Figure S11). After 83 days of storage in inert atmosphere, the solar cells retained 92%, and after 184 days about 76% of their initial PCE.

The EQE spectra (Fig. 4b) show a broad photoresponse from 450 to 800 nm with a maximum at ca. 700 nm. The shapes of the EQE spectra are in good agreement with the optical absorption properties of the polymer and the acceptor. The region between 450 and 600 nm is mainly governed by the absorption of the conjugated polymer, while the photocurrent generated in the wavelength region between 650 and 800 nm originates from the contribution of the NFA (Figure S5). The cumulated *J*_SC_ values of 5.30 mA cm^−2^ (ZnO, inverted) and 5.21 mA cm^−2^ (PEDOT–PSS, normal) correlate well with the measured *J*_SC_ (5.46 mA cm^−2^ for ZnO, 4.92 mA cm^−2^ for PEDOT–PSS) using a shadow mask (2.65 × 2.65 mm).

Furthermore, there is potential for improving the photovoltaic performance of the polymer since the good solubility of PTz4T-2OD would allow preparing batches with a higher molecular weight, while maintaining good processability. Increased molecular weight has been identified as a key to improving the efficiency of polymer-based solar cells [32, 36]. To further optimize the material, diligent purification steps, including preparative GPC to selectively isolate high molecular weight fractions with a low polydispersity, are required and are expected to further push the performance towards higher PCEs [37].

## Conclusions

The synthesized conjugated tetrazine–quaterthiophene copolymer PTz4T-2OD features a band gap of 1.8 eV, an absorption coefficient of up to 1.9 × 10^5^ cm^−1^ and a good solubility with a molecular weight (*M*_n_) of 24.1 kDa. In thin films, the polymer reveals a preferred face-on orientation with respect to the substrate and a smooth surface morphology. Moreover, a good thermal stability was observed up to 288 °C, the temperature at which the tetrazine rings in the backbone start to decompose.

The photovoltaic performance of PTz4T-2OD was investigated in organic solar cells in combination with the non-fullerene acceptor ITIC-F in inverted and normal device configurations. PTz4T-2OD and ITIC-F have complementary absorption properties, and the EQE spectra confirm a contribution of both materials to the photocurrent generation in a wavelength range between 400 and 800 nm. The solar cells showed high open-circuit voltages of approx. 900 mV; however, the photocurrents (6–7 mA cm^−2^) and FF values (40–44%) remained limited. The highest power conversion efficiencies of 2.6% were obtained in the inverted solar cell architecture.

## Electronic supplementary material

Below is the link to the electronic supplementary material.

Supplementary material 1 (DOCX 1345 kb)
