# Supplementary material for: Synthesis of a tetrazine–quaterthiophene copolymer and its optical, structural and photovoltaic properties
Source: J Mater Sci. 2019 Apr 3;54(13):10065–76. doi: 10.1007/s10853-019-03551-3 (PMC6472551; doi:10.1007/s10853-019-03551-3)
Supplement: Supplementary file 1 — Supplementary material 1 (DOCX 1345 kb) [file 10853_2019_3551_MOESM1_ESM.docx]

From: Rath, Thomas <thomas.rath@tugraz.at>
Sent: 31 March 2019 17:28
To: Subhashini Gopal
Subject: AW: Proofs for your article in JOURNAL OF MATERIALS SCIENCE (3551)
Attachments: Proof_Corrections_Article_10853_2019_3551.docx; Figure_2_new.jpg; Figure_2_new_EMF-file.emf

Dear Springer Correction Team,

I send you the proof corrections of article 10853_2019_3551 (and also the new Figure 2) attached to this email.

Please contact me, if you have any questions regarding the corrections.

Thank you very much and best regards,
Thomas Rath

--
Dr. Thomas Rath
Institute for Chemistry and Technology of Materials (ICTM) Graz University of Technology Stremayrgasse 9/V
A-8010 Graz
phone: 004331687332281

Von: spr_corrections2@springer.com <spr_corrections2@springer.com>
Gesendet: Dienstag, 26. März 2019 14:05:14
An: Rath, Thomas
Betreff: Proofs for your article in JOURNAL OF MATERIALS SCIENCE (3551)

Article Title: SYNTHESIS OF A TETRAZINE-QUATERTHIOPHENE COPOLYMER AND ITS OPTICAL, STRUCTURAL AND PHOTOVOLTAIC PROPERTIES
DOI: 10.1007/s10853-019-03551-3
Editorial manuscript number: JMSC-D-19-01000.1

Dear Author,

We are pleased to inform you that your paper is nearing publication. The page proofs are available at:

https://www.e-proof.sps.co.in/springer/ja.asp?rfp=authfcerfbcengu

The URL is valid only until your paper is published online. It is for proof purposes only and may not be used by third parties.

The proof shows the paper as it will appear later in print except that:

. The pages are not numbered but the lines are, to ease reference to any passage to be corrected.
. This proof has been optimized for online presentation.Images will be printed in color.
. This article will appear in Springer's Open Choice program and will be made available with full open access.

You can help us facilitate rapid publication by returning the corrected proof of this paper within 2

working days. Please first read about the proof procedure to learn how to proceed and also to obtain information about online publication.

Please select three (or fewer) keywords for your paper from the list which is enclosed with the proof link and return this to Springer Author Correction team at (spr_corrections2@springer.com)

Please ensure you fill out your response to the AUTHOR QUERIES raised (if any) during the process of typesetting and return this form along with your corrections. Without your response to these queries, we may not be able to continue with the processing of your article for Online Publication.

In case of difficulties with the proofs, please contact me.

Thank you very much. We hope you are pleased with the publication.

Sincerely yours,

Springer Correction Team

No. 6&7, 5th Street, Radhakrishnan Salai Mylapore, Chennai, Tamilnadu India, Pincode 600 004
e-mail: spr_corrections2@sps.co.in
Fax: +91 73 0588 0700 (or) +91 44 4208 9499

This e-mail is confidential and should not be used by anyone who is not the original intended recipient. If you have received this e-mail in error please inform the sender and delete it from your mailbox or any other storage mechanism. Scientific Publishing Services Private Limited does not accept liability for any statements made which are clearly the sender's own and not expressly made on behalf of Scientific Publishing Services Private Limited or one of their agents.
Please note that Scientific Publishing Services Private Limited and their agents and affiliates do not accept any responsibility for viruses or malware that may be contained in this e-mail or its attachments and it is your responsibility to scan the e-mail and attachments (if any).
Scientific Publishing Services Private Limited. Registered office: No. 6 & 7, 5th Street, R.K.Salai, Mylapore, Chennai, 600004, India.
Registered number: U22219TN1992PTC022318

Proof Corrections for Article 10853_2019_3551

Queries:

AQ1. The author names are correct.

AQ2. The edit made in the title is correct.

AQ3. The corresponding author email is correct.

AQ4. The reference citation is correct.

AQ5. Ref 1: This paper only has an article number, no start and end pages.

Ref 8: This paper only has an article number, no start and end pages.

Ref 13: Please change the year of this reference from "2018" to "2019". This paper only has an article number, no start and end pages.

Ref 16: This paper only has an article number, no start and end pages.

Ref 17: This paper only has an article number, no start and end pages.

Keywords:

Organic solar –cell materials; polymers; photovoltaics (solar cells)

Researcher information – Please add the following ORCID-ID:

Sven Noesberger: 0000-0002-4025-9151

Corrections:

28: please change "catalysed" to "catalyzed"

74: Please change: "One example for such an acceptor building block is tetrazines" to "One example for such acceptor building blocks are tetrazines"

126-128: Please change:

"poly[3-(3-(2-octyldodecyl)-[2,2':5',2''-quaterthiophen]-5-yl)-6-(4-(2-octyldodecyl)thiophen-2-yl-1,2,4,5-tetrazine]"

to

"poly[(1,2,4,5-tetrazin-3,6-diyl)-*alt*-(3,3'''-di(2-octyldodecyl)-2,2';5',2'';5'',2'''-quaterthiophen-5,5'''-diyl)]"

154: please change "5'-bis(trimethylstannyl)-2,2'-bithiophene" to "5,5'-bis(trimethylstannyl)-2,2'-bithiophene"

156: Please change "(o-tol)$_3$P" to "P(o-tol)$_3$"

163: Please change "programme" to "program"

178: Please change "FT-IR: (cm^{-1}):" to "FT-IR (cm^{-1}):"

192: Please change "using undoped Si-wafer as substrate" to "using undoped Si-wafers as substrates"

220: Please change "flow rate 50 mL min^{-1}" to "flow rate: 50 mL min^{-1}"

236: Please write Bu_4NPF_6 in one line. This chemical formula should not be divided.

237: Please change "and a scan rate 50 mV s^{-1}" to "and a scan rate of 50 mV s^{-1}."

249/250: The line break is not necessary here, please remove it.

291: Please change "0.45-mm" to "0.45-µm"

347: Please change "5-bromo-4-(-2-octyl…)" to "5-bromo-4-(2-octyl…) "

373-375: Please change:

"poly[3-(3-(2-octyldodecyl)-[2,2':5',2''-quaterthiophen]-5-yl)-6-(4-(2-octyldodecyl)thiophen-2-yl-1,2,4,5-tetrazine]"

to

"poly[(1,2,4,5-tetrazin-3,6-diyl)-*alt*-(3,3'''-di(2-octyldodecyl)-2,2';5',2'';5'',2'''-quaterthiophen-5,5'''-diyl)]"

377: Please write $Pd_2(dba)_3$ in one line. This chemical formula should not be divided.

494: "S_{q-}": the "$_-$" is not necessary here. It should read "(S_q = 1.5 nm)"

Figure 2: We changed "Insnsity" to "Intensity" in Figure 2B. Please change Figure 2 to the new one below. A jpg-file and an emf-file of this figure are also provided with this email.

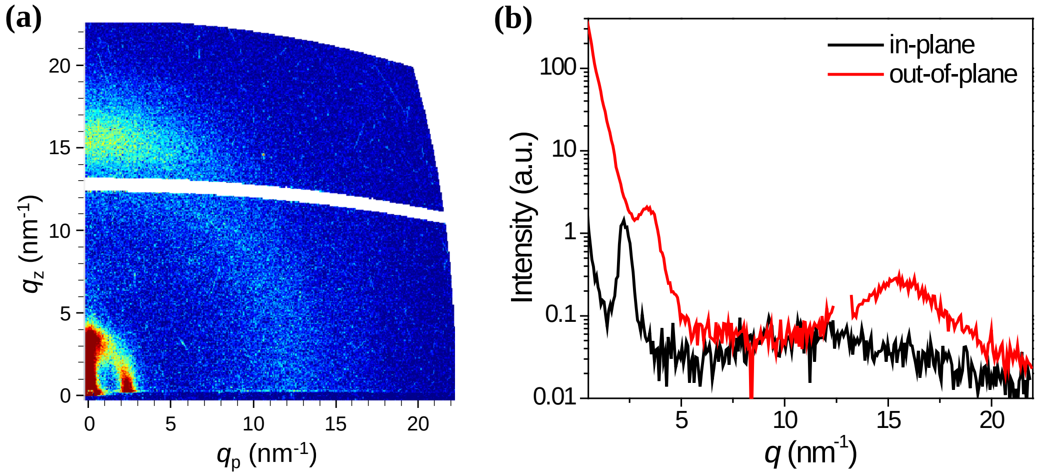

Caption of Figure 4b: please change “EQE spectrum” to “EQE spectra”

547: Please change “76%, of their” to “76% of their”

581: Please change “24.6 kDa” to “24.1 kDa”

583: Please change “… and smooth surface …” to “… and a smooth surface …”

592: Please change “of both to” to “of both materials to”

References:

Ref 4: Please change “Chenxi L” to “Li C”

Ref 6: Please change “Houa J, Wanga Z” to “Hou J, Wang Z”

Ref 9: The page numbers of this article are now available. Please change the last line of this reference to: “Chem Soc Rev 48:1596-1625

Ref 18: Please change “Suman Bagui A,” to “Suman, Bagui A,”

Please contact me, if you have any questions regarding the corrections.

Thank you very much and best regards,
Thomas Rath

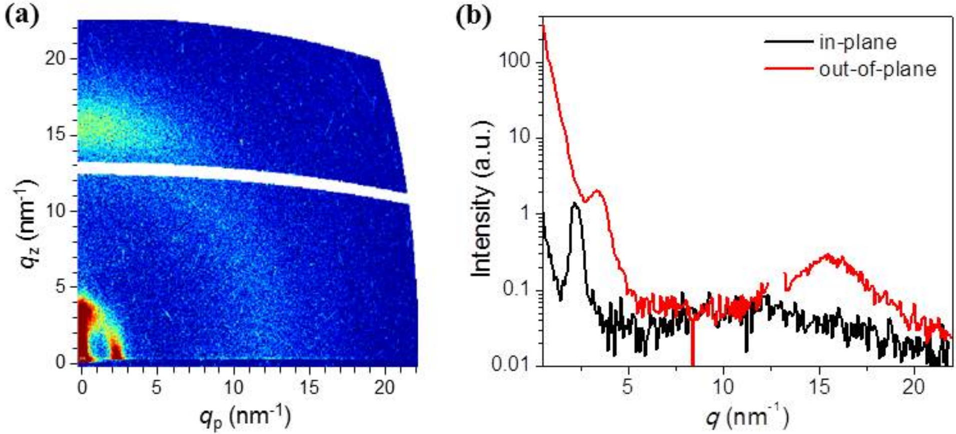

(a)
q_z (nm^{-1})
q_p (nm^{-1})
0
5
10
15
20
(b)
in-plane
out-of-plane
Intensity (a.u.)
q (nm^{-1})
0.01
0.1
1
10
100
5
10
15
20

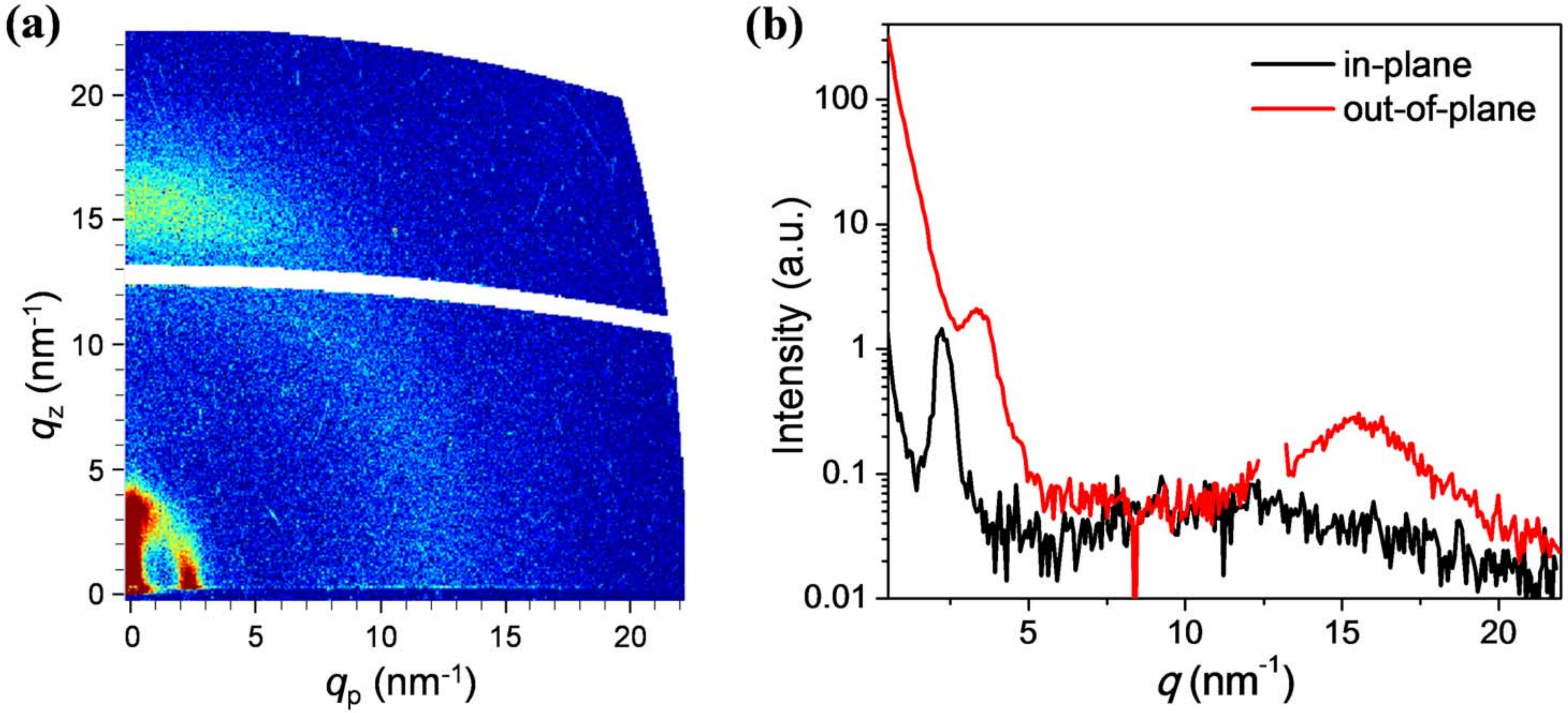
(a)
q_z (nm^{-1})
q_p (nm^{-1})
0
5
10
15
20
(b)
in-plane
out-of-plane
Intensity (a.u.)
q (nm^{-1})
0.01
0.1
1
10
100
5
10
15
20
